# Conversion Surgery Following Durvalumab Plus Tremelimumab in Unresectable Hepatocellular Carcinoma with Para-Aortic Lymph Node Metastases: A Case of Complete Hepatic Response and Lymphatic Resistance

**DOI:** 10.70352/scrj.cr.25-0539

**Published:** 2025-12-13

**Authors:** Sunao Uemura, Teppei Tokumaru, Shuta Tamura, Motoyasu Tabuchi, Shinya Sakamoto, Rika Yoshimatsu, Manabu Matsumoto, Jun Iwata, Takehiro Okabayashi

**Affiliations:** 1 Department of Gastroenterological Surgery, Kochi Health Sciences Center, Kochi, Kochi, Japan; 2 Department of Radiology, Kochi Health Sciences Center, Kochi, Kochi, Japan; 3 Department of Diagnostic Pathology, Kochi Health Sciences Center, Kochi, Kochi, Japan

**Keywords:** conversion surgery, unresectable hepatocellular carcinoma, immune checkpoint inhibitor, para-aortic lymph node metastasis, undifferentiated carcinoma component

## Abstract

**INTRODUCTION:**

Recent advances in immune checkpoint inhibitor (ICI) therapy have made conversion surgery possible in selected patients with unresectable hepatocellular carcinoma (uHCC); however, outcomes in cases with extrahepatic spread remain unclear.

**CASE PRESENTATION:**

A 77-year-old man with hepatitis B-associated uHCC presented with a 60 mm tumor in the posterior section of the liver with several daughter nodules and multiple enlarged para-aortic lymph nodes (PALNs). Durvalumab plus tremelimumab was chosen over atezolizumab plus bevacizumab because of the patient’s concurrent anticoagulant therapy for atrial fibrillation, which raised concerns about bleeding risk. Tumor markers (alpha-fetoprotein and protein induced by vitamin K absence or antagonist-II) returned to normal after three cycles, and imaging demonstrated significant shrinkage of both the primary tumor and the lymph nodes. After nine treatment cycles, the primary tumor had further decreased to 25 mm without daughter nodules; however, two PALNs demonstrated regrowth. The patient subsequently underwent a subsegmentectomy of segment 6 and lymphadenectomy of the PALNs. Histopathological examination revealed a complete pathological response (pCR) in the liver tumor, with no viable tumor cells. However, metastatic lymph nodes contained viable undifferentiated carcinoma cells that were negative for hepatocellular markers (HepPar-1 and arginase-1) but positive for CAM5.2 and vimentin, indicating a treatment-resistant component. Twelve months postoperatively, the patient remained recurrence-free with preserved liver function and good general condition.

**CONCLUSIONS:**

This case demonstrates a discrepant therapeutic response, with a pCR in the liver contrasted with residual undifferentiated carcinoma in the PALNs. To our knowledge, this is the first reported HCC case demonstrating hepatic pCR after ICI therapy with residual undifferentiated carcinoma confined to the PALNs, highlighting immune-resistant extrahepatic clones and the diagnostic–therapeutic value of conversion surgery.

## Abbreviations

Dur
: durvalumab
EMT
: epithelial–mesenchymal transition
HCC
: hepatocellular carcinoma
ICI
: immune checkpoint inhibitor
PALN
: para-aortic lymph node
Tre
: tremelimumab

## INTRODUCTION

HCC, the most common primary liver malignancy, represents a major global health burden and ranks among the leading causes of cancer-related mortality worldwide.^1)^ Despite improved surveillance strategies in high-risk populations, many patients are still diagnosed at advanced stages, often presenting with vascular invasion or extrahepatic spread that renders them ineligible for curative therapies such as surgical resection or liver transplantation.^1,2)^ Consequently, systemic therapy remains the mainstay of treatment for patients with unresectable HCC (uHCC).^2)^

ICIs have transformed the therapeutic landscape of uHCC. The IMbrave150 trial demonstrated that the combination of atezolizumab and bevacizumab (Atezo/Bev) significantly improved overall survival (OS) and progression-free survival compared with sorafenib, thereby establishing Atezo/Bev as a 1st-line standard of care.^3)^ Subsequently, the HIMALAYA trial introduced the STRIDE regimen, which consists of a single priming dose of Tre plus Dur, followed by Dur monotherapy, which demonstrated a significant OS benefit with an acceptable safety profile.^4)^ These ICI-based regimens have not only improved response rates but have also opened new avenues for downstaging advanced tumors, enabling potential conversion to curative therapies.

As systemic therapy continues to evolve, increasing attention has been directed toward conversion surgery, defined as curative-intent resection following substantial tumor downstaging achieved with systemic treatment.^5)^ Reports of successful conversion surgery after combination immunotherapy are emerging in patients with initially unresectable disease, including those with macrovascular invasion or extrahepatic metastases.^5)^ However, clinical evidence remains limited, and the histopathological characteristics of residual or regrown lesions following immunotherapy are not yet fully understood.

We report a rare and informative case of a 77-year-old man with uHCC and PALN metastases who achieved a complete pathological response (pCR) in the primary liver tumor after treatment with the STRIDE regimen. Despite regrowth of the PALN lesions, surgical resection was performed safely and revealed viable undifferentiated carcinoma components in the lymph nodes. Notably, we report a paradoxical response pattern—hepatic pCR with residual undifferentiated PALN disease after STRIDE—that has not been previously described in the HCC literature. This case underscores the dual role of conversion surgery, not only in achieving therapeutic benefit in selected patients but also in providing unique pathological insights into resistant tumor biology.

## CASE PRESENTATION

**Fig. 1:**
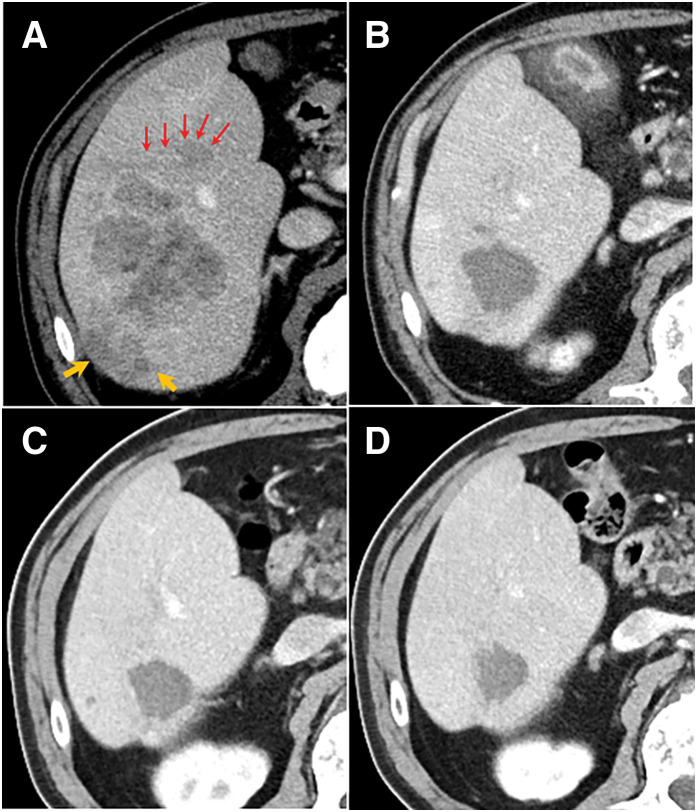
Radiological response of the primary hepatic tumor. (**A**) Pre-treatment contrast-enhanced abdominal CT showing a large, diffuse tumor (maximum diameter: 60 mm) in the posterior segment of the right hepatic lobe, with multiple daughter nodules (orange arrows) and invasion of the Glissonean branch of segment 6a (red arrows). (**B**) After three cycles of the STRIDE regimen, the hepatic tumor had shrunk significantly to 30 mm. (**C**) After six cycles, the primary tumor remained stable in size, and the daughter nodules became indistinct. (**D**) After nine cycles, the hepatic tumor had further shrunk to 25 mm, with complete disappearance of the daughter nodules.

**Fig. 2:**
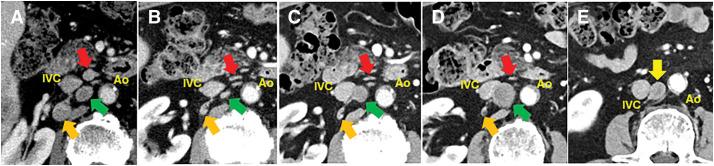
Radiological response of PALNs. (**A**) Pre-treatment contrast-enhanced abdominal CT showing multiple enlarged PALNs: 9 × 9 mm at the 16b1 interaortocaval region (red arrow), 15 × 10 mm at the 16b2 retrocaval region (orange arrow), and 14 × 12 mm at another 16b2 retrocaval site (green arrow). (**B**) After three cycles of the STRIDE regimen, the PALNs had decreased in size. (**C**) After six cycles, one previously regressed lymph node (green arrow) showed signs of regrowth. (**D**) After nine cycles, the same lymph node had enlarged to 15 × 15 mm (green arrow), whereas the other PALNs (red and orange arrows) remained reduced in size. (**E**) A newly enlarged 16b retrocaval lymph node measuring 15 × 12 mm appeared (yellow arrow). Ao, aorta; IVC, inferior vena cava; PALN, para-aortic lymph node

A 77-year-old man presented to a local hospital with fever and right abdominal pain. His medical history included chronic hepatitis B virus infection, hypertension, and atrial fibrillation, for which he was receiving anticoagulation therapy. Laboratory tests revealed inflammatory findings with elevated biliary marker, raising suspicion for hepatobiliary disease. CT revealed a liver tumor, and the patient was referred to our hospital for further evaluation. At our institution, inflammatory findings were improving, liver and renal functions were within normal limits, and the prothrombin time was 31%, prolonged due to anticoagulation therapy. Tumor markers were markedly elevated: alpha-fetoprotein (AFP) of 1243.6 ng/mL (normal: 0–10.0 ng/mL) and protein induced by vitamin K absence or antagonist-II (PIVKA-II) of 809 mAU/mL (normal: 0–39 mAU/mL). Contrast-enhanced abdominal CT revealed a large, diffuse tumor measuring up to 60 mm in the posterior segment of the right hepatic lobe, with several daughter nodules and invasion of the Glissonean branch of segment 6a (**Fig. 1A**). No distant organ metastases were detected; however, multiple PALNs measured 9 × 9 mm in the 16b1 interaortocaval region, 15 × 10 mm in the 16b2 retrocaval region, and 14 × 12 mm in another 16b2 retrocaval node (**Fig. 2A**). No other primary origins of lymphadenopathy were identified. The tumor was diagnosed as a uHCC with multiple PALN metastases, corresponding to the Barcelona Clinic Liver Cancer stage C.

According to the 2021 guidelines of the Japanese Society of Hepatology, systemic therapy was indicated because of preserved liver function (Child–Pugh score, 6; grade A). The patient received combination immunotherapy with Tre (300 mg) and Dur (1500 mg), followed by maintenance Dur (1500 mg), in accordance with the STRIDE regimen from the HIMALAYA trial.^3)^ After three cycles, AFP and PIVKA-II levels normalized. CT revealed significant tumor shrinkage to 30 mm (**Fig. 1B**). The PALNs also decreased in size (**Fig. 2B**), and the overall response was classified as a partial response according to the RECIST criteria. After six cycles, tumor marker levels remained within normal limits, the primary tumor size was stable, and the daughter nodules had become indistinct (**Fig. 1C**). However, one previously regressed PALN showed signs of regrowth (**Fig. 2C**). After nine treatment cycles, the primary tumor had further decreased to 25 mm and no longer exhibited daughter nodules (**Fig. 1D**); however, the same lymph node had enlarged to 15 × 15 mm (**Fig. 2D**). Two other previously enlarged lymph nodes remained decreased in size, whereas a new 16b retrocaval lymph node measured 15 × 12 mm (**Fig. 2E**). The disease was therefore reclassified as progressive disease (PD) based on RECIST. The patient experienced no immune-related adverse events or deterioration in liver function during the treatment period.

After nine cycles of Dur/Tre (approximately nine months after treatment initiation), the hepatic lesion had achieved a radiological complete response with sustained tumor marker normalization. However, two PALNs showed radiological regrowth, and the disease was reclassified as PD according to RECIST criteria. At this point, the treating team considered alternative systemic therapy options, including switching to another ICI regimen, but efficacy as a second-line therapy remained unproven and raised concerns for further clonal selection under immune pressure. Therefore, systemic therapy was discontinued after the ninth cycle, and conversion surgery was selected to achieve curative resection of all visible disease and to eliminate resistant clones confined to the PALNs. Initially, the tumor occupied the entire posterior section of the liver, but post-treatment imaging showed localization to segment 6 (S6) with the disappearance of daughter nodules. Given the prior evidence of vascular invasion into the Glissonean branch of segment 6a (G6a), an anatomical subsegmentectomy of S6 was planned. Residual liver functional assessments (remKICG and remKGSA) exceeded safety thresholds, supporting an anatomical S6 subsegmentectomy to preserve postoperative liver function, with systematic lymphadenectomy of the involved 16b1/16b2 regions. Surgery was performed nine months after treatment initiation and three weeks after the final Dur dose. The operative time was 287 min, with total blood loss of 425 mL. No intraoperative blood transfusions were required. The postoperative course was uneventful, and the patient was discharged on POD 8 in good condition.

**Fig. 3:**
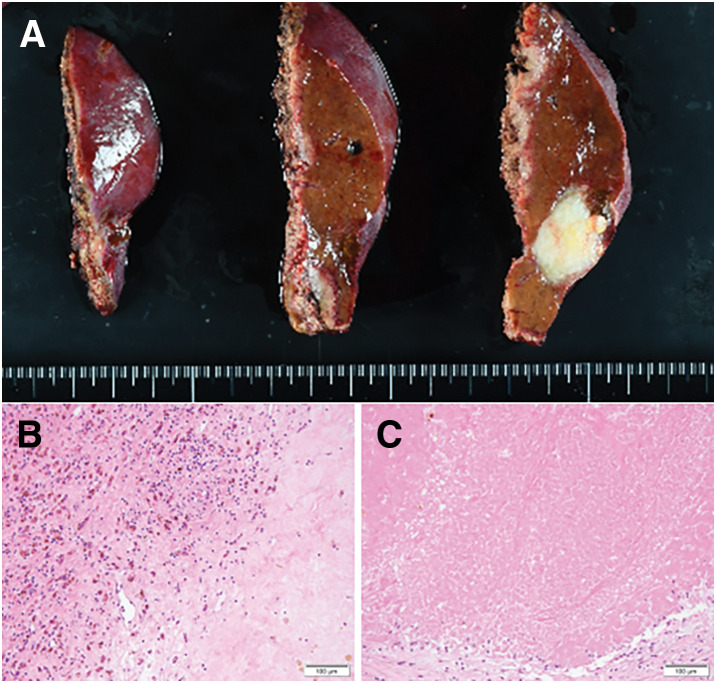
Resected specimen and histopathological findings of the hepatic tumor. (**A**) Macroscopic examination of the resected specimen showing a whitish nodule measuring 23 × 17 mm. (**B**, **C**) Hematoxylin and eosin staining of the nodule revealed fibrotic nests without viable tumor cells, consistent with a complete pathological response to preoperative therapy.

**Fig. 4:**
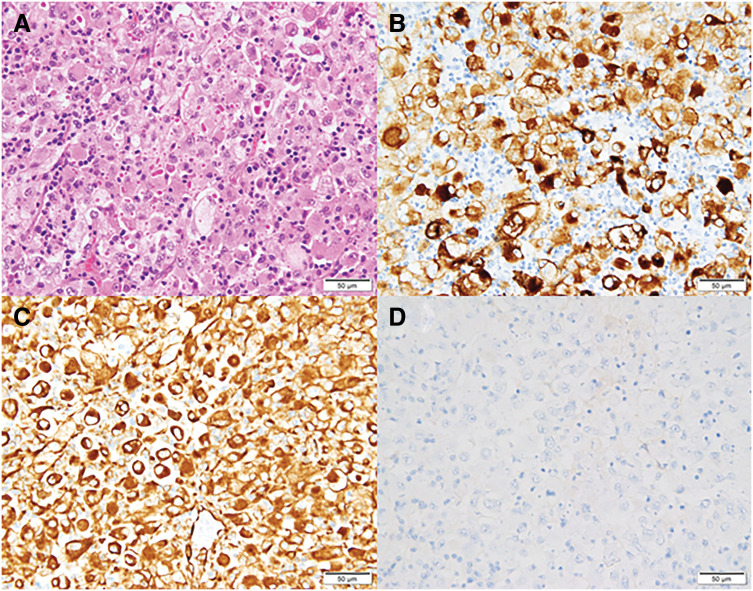
Histopathological findings of para-aortic lymph node metastases. (**A**) Hematoxylin and eosin staining revealed round atypical cells with irregular nuclei, eosinophilic cytoplasm, and prominent neutrophilic infiltration. (**B**) Immunohistochemistry showing positive staining for CAM5.2. (**C**) Positive staining for vimentin. (**D**) Negative staining for E-cadherin.

Macroscopically, the residual liver tumor was a well-demarcated, whitish lesion measuring 23 × 17 mm (**Fig. 3A**). Histological examination revealed fibrotic nests without viable tumor cells, consistent with a pCR to the preoperative therapy (**Fig. 3B** and **3C**). The background liver exhibited features of chronic hepatitis B, including portal area expansion, portal–portal bridging fibrosis, and a METAVIR score of A1/F2. Metastatic involvement was identified in two PALNs, corresponding to the lymph nodes noted as enlarged on preoperative imaging. These lymph nodes were infiltrated by round atypical cells with irregular nuclei, eosinophilic cytoplasm, and prominent neutrophilic infiltrates (**Fig. 4A**). The presence of poorly cohesive, dysmorphic cells suggested a partial treatment effect. Immunohistochemical staining was positive for CAM5.2 and vimentin (**Fig. 4B** and **4C**) but negative for hepatocyte-specific antigen, glypican-3, arginase-1, and AFP. Additional staining was negative for CD34, CD68, α-smooth muscle actin, Melan A, and E-cadherin (**Fig. 4D**), suggestive of EMT-like features. The morphology and immunophenotype were consistent with undifferentiated carcinoma, although EMT-mediated phenotypic changes in metastatic HCC cells could not be excluded. However, the complete absence of hepatocellular markers (HepPar-1, glypican-3, arginase-1, and AFP), combined with strong CAM5.2 and vimentin expression and the absence of trabecular or pseudoacinar architecture, argued against poorly differentiated HCC.

At the 12-month follow-up, the patient remained recurrence-free and in good general condition based on physical examinations, tumor marker evaluations, and contrast-enhanced CT scans conducted every three months.

## DISCUSSION

In recent years, systemic therapy has transformed the treatment landscape for uHCC, offering new opportunities for conversion surgery. Immunotherapy, particularly ICIs, has demonstrated significant efficacy in inducing tumor regression in selected patients, thereby enabling surgical resection in cases previously deemed inoperable.^5)^ Both the IMbrave150 and HIMALAYA trials showed that combination regimens such as Atezo/Bev and Dur plus Tre (Dur/Tre) significantly improved OS in patients with advanced HCC.^2,3)^

In our case, the choice of Dur/Tre over Atezo/Bev was based on the patient’s need for anticoagulation therapy for atrial fibrillation. Treatment with bevacizumab is associated with an increased risk of bleeding complications, particularly in patients with portal hypertension or coagulopathy.^2)^ Dur/Tre, which lacks antiangiogenic components, was therefore considered a safer option in this clinical context and has been validated as a first-line treatment for advanced HCC in the HIMALAYA trial.^3)^ At the time of this case, only Atezo/Bev and Dur/Tre were approved in Japan. At present, nivolumab plus ipilimumab (Ipi/Nivo) has also been approved^6)^; however, its superiority over STRIDE remains unproven, and its higher incidence of immune-related adverse events warrants caution, particularly in elderly or comorbid patients. In this case, Dur/Tre was fortunately well tolerated without notable toxicity. Following Dur/Tre therapy, the patient showed a marked response, with normalization of tumor markers and radiologic regression of both the primary hepatic tumor and PALN metastases. However, after initial disease control, regrowth of the PALNs was observed, prompting surgical resection. An alternative approach could have been to switch from Dur/Tre to Ipi/Nivo, had it been available. However, its efficacy as a second-line option remains uncertain, and in this case, progression was confined to the PALNs after a complete hepatic response. Continued systemic therapy risked further clonal evolution, whereas surgical resection allowed complete removal of all visible disease. Thus, conversion surgery was considered more appropriate, although this remains a speculative interpretation. Pathologic examination revealed a complete response in the liver tumor, but viable tumor cells persisted in the lymph nodes. Interestingly, these residual cells lacked hepatocellular markers and instead expressed CAM5.2 and vimentin, consistent with an undifferentiated phenotype.

To our knowledge, this is the first reported case of HCC demonstrating a pCR in the liver after immunotherapy while harboring residual undifferentiated carcinoma in the PALNs, which further underscores the novelty of our report. This finding highlights the potential for immune-resistant tumor clones to persist in extrahepatic sites despite the apparent eradication of the primary tumor. This discrepancy underscores the heterogeneity of HCC and suggests that conversion surgery can provide not only therapeutic benefit but also pathological insight into resistant clones. These findings underscore the biological complexity of uHCC, particularly in cases with extrahepatic disease. The coexistence of treatment-sensitive and treatment-resistant tumor clones reflects the well-recognized heterogeneity of HCC. Intratumoral heterogeneity influences the immune microenvironment and the response to ICIs.^7)^ Clonal selection under therapeutic pressure can lead to the expansion of dedifferentiated or mesenchymal-like cells, facilitating immune escape by downregulating antigen presentation and modulating cytokine signaling.^8,9)^ EMT in resistant tumor components is of particular interest, as EMT contributes to the acquisition of stem-like properties and immune resistance and has been implicated in tumor progression and metastasis in HCC.^10)^ In our case, the expression of vimentin and the loss of hepatocyte-specific antigens in the metastatic lymph nodes suggested EMT and dedifferentiation, supporting the hypothesis that immune-resistant subpopulations persisted despite systemic therapy.^10,11)^ Furthermore, immune selection pressure exerted by ICIs may facilitate the survival and clonal expansion of poorly differentiated, therapy-resistant cells. Such selection may have allowed mesenchymal-like or undifferentiated clones to evade immune surveillance, ultimately manifesting as the regrowth of metastatic lesions despite initial tumor control. It remains unclear whether the EMT-like phenotype in the metastatic lymph nodes was pre-existing or induced under immune pressure. Recent studies in lung and pancreatic cancers have demonstrated that EMT contributes to resistance against ICI therapy.^12,13)^ In our case, the initial regression of the affected lymph node followed by subsequent regrowth suggests that EMT was likely induced or enhanced during treatment rather than solely reflecting a baseline phenotype. However, in the absence of pretreatment pathological specimens, this cannot be definitively established and warrants further investigation.

A recent study by Chan et al. demonstrated that early-onset HCC can exhibit aggressive behavior characterized by enhanced Notch signaling and an undifferentiated, stem-like phenotype.^14)^ These molecular features promote both tumor initiation and immunogenicity, potentially rendering such tumors more responsive to ICIs. In our case, the undifferentiated histology of the metastatic lymph nodes, combined with the complete response in the liver, may reflect heterogeneous tumor biology in which ICI-sensitive and ICI-resistant clones coexist. The primary hepatic lesion may have harbored immunologically “hot” features conducive to immune-mediated clearance, whereas the metastatic lymph nodes may have represented a subpopulation that escaped immune surveillance, possibly through phenotypic plasticity or immune-evasion mechanisms such as EMT. This observation underscores the need for deeper molecular profiling of the residual disease after ICI therapy to better understand the differential responses across tumor compartments.

Conversion surgery for uHCC with extrahepatic spread, particularly PALN metastasis, remains a subject of debate. PALN involvement is typically considered indicative of systemic disease and is associated with a poor prognosis. However, emerging evidence suggests favorable outcomes in selected patients undergoing resection after a good response to systemic therapy.^5)^ The optimal timing for conversion surgery remains an open question. Delayed intervention may allow systemic therapy more time to induce further tumor regression and to identify long-term responders, but it also carries the risk of progression of resistant clones, as observed in our case. Conversely, early surgery after initial tumor shrinkage may prevent regrowth, but it risks an insufficient pathological response or residual micrometastasis. In our patient, surgery was performed after nine cycles (ninth months), at which point a resistant clone had reemerged. This finding suggests that prolonged treatment may permit the emergence of resistant subclones and underscores the importance of individualizing surgical timing using dynamic imaging, tumor markers, and patient fitness.

Recent evidence on the optimal timing of conversion surgery for HCC, particularly after immunotherapy, highlights tumor marker normalization as a critical parameter. Clinical complete response, defined as sustained radiological complete response and tumor marker normalization (AFP <7 ng/mL and PIVKA-II <40 mAU/mL) for at least four weeks, has been identified as a strong predictor of pathological complete response in recent studies.^15)^ In our case, although the tumor markers normalized early, there was regrowth of the PALN during treatment, indicating the emergence of resistant tumor clones. The association between EMT and treatment resistance was a key insight in our case. In HCC, EMT is characterized by vimentin-expressing cells, as observed in metastatic lymph nodes, and by immunosuppressive changes in the tumor microenvironment.^16,17)^ Phenotypic changes in tumor cells associated with EMT can alter the expression of immune checkpoint molecules such as PD-L1 and CTLA-4, thereby promoting resistance to immunotherapy.^18)^ The mesenchymal marker expression pattern in our case was consistent with EMT, suggesting resistance to Dur and Tre compared with conventional HCC.^19)^

In the present case, conversion surgery enabled complete resection of all visible disease, and pathological evaluation revealed residual tumors only in the lymph nodes. The discrepancy between radiological findings and pathological responses further emphasizes the limitations of imaging in assessing post-immunotherapy disease status.^13)^ Therefore, surgical intervention offers not only therapeutic benefits but also valuable pathological information on the nature of residual disease. This case highlights the need for long-term surveillance after conversion surgery, particularly when undifferentiated or EMT-like tumor features are present. The clinical implications of these resistant subclones are not yet fully understood but may portend recurrence or metastasis, necessitating vigilant follow-up and consideration of adjuvant strategies. Further studies are warranted to elucidate mechanisms of immune resistance and to optimize the timing and patient selection for conversion surgery.

## CONCLUSIONS

This case demonstrates a complete hepatic pathological response with residual undifferentiated PALN disease after ICI therapy, implicating extrahepatic resistant subclones—potentially EMT-related. Conversion surgery may offer oncologic control and pathological insight, supporting vigilant surveillance and further study of resistance and surgical timing.

## DECLARATIONS

### Availability of data and materials

All data generated or analyzed during this study are included in the published article.

## References

[1] Bray F, Laversanne M, Sung H, et al. Global cancer statistics 2022: GLOBOCAN estimates of incidence and mortality worldwide for 36 cancers in 185 countries. CA Cancer J Clin 2024; 74: 229–63. 38572751 10.3322/caac.21834

[2] Gordan JD, Kennedy EB, Abou-Alfa GK, et al. Systemic Therapy for Advanced Hepatocellular Carcinoma: ASCO Guideline Update. J Clin Oncol 2024; 42: 1830–50. 38502889 10.1200/JCO.23.02745

[3] Finn RS, Qin S, Ikeda M, et al. Atezolizumab plus Bevacizumab in Unresectable Hepatocellular Carcinoma. N Engl J Med 2020; 382: 1894–905. 32402160 10.1056/NEJMoa1915745

[4] Abou-Alfa GK, Lau G, Kudo M, et al. Tremelimumab plus Durvalumab in Unresectable Hepatocellular Carcinoma. NEJM Evid 2022; 1: EVIDoa2100070. 38319892 10.1056/EVIDoa2100070

[5] Arita J, Ichida A, Nagata R, et al. Conversion surgery after preoperative therapy for advanced hepatocellular carcinoma in the era of molecular targeted therapy and immune checkpoint inhibitors. J Hepatobiliary Pancreat Sci 2022; 29: 732–40. 35306748 10.1002/jhbp.1135

[6] Yau T, Galle PR, Decaens T, et al. Nivolumab plus ipilimumab versus lenvatinib or sorafenib as first-line treatment for unresectable hepatocellular carcinoma (CheckMate 9DW): an open-label, randomised, phase 3 trial. Lancet 2025; 405: 1851–64. 40349714 10.1016/S0140-6736(25)00403-9

[7] Vitale I, Shema E, Loi S, et al. Intratumoral heterogeneity in cancer progression and response to immunotherapy. Nat Med 2021; 27: 212–24. 33574607 10.1038/s41591-021-01233-9

[8] Pérez-González A, Bévant K, Blanpain C. Cancer cell plasticity during tumor progression, metastasis and response to therapy. Nat Cancer 2023; 4: 1063–82. 37537300 10.1038/s43018-023-00595-y PMC7615147

[9] Sayaman RW, Saad M, Thorsson V, et al. Germline genetic contribution to the immune landscape of cancer. Immunity 2021; 54: 367–86.e8. 33567262 10.1016/j.immuni.2021.01.011 PMC8414660

[10] Jayachandran A, Dhungel B, Steel JC. Epithelial-to-mesenchymal plasticity of cancer stem cells: therapeutic targets in hepatocellular carcinoma. J Hematol Oncol 2016; 9: 74. 27578206 10.1186/s13045-016-0307-9 PMC5006452

[11] Park MY, Kim KR, Park HS, et al. Expression of the serum response factor in hepatocellular carcinoma: implications for epithelial-mesenchymal transition. Int J Oncol 2007; 31: 1309–15. 17982656

[12] Furuhashi S, Morita Y, Matsumoto A, et al. Tenascin C in pancreatic cancer-associated fibroblasts enhances epithelial mesenchymal transition and is associated with resistance to immune checkpoint inhibitor. Am J Cancer Res 2023; 13: 5641–55. 38058842 PMC10695794

[13] Jeong H, Koh J, Kim S, et al. Epithelial-mesenchymal transition induced by tumor cell-intrinsic PD-L1 signaling predicts a poor response to immune checkpoint inhibitors in PD-L1-high lung cancer. Br J Cancer 2024; 131: 23–36. 38729997 10.1038/s41416-024-02698-4 PMC11231337

[14] Chan ASL, Zhu H, Narita M, et al. Titration of RAS alters senescent state and influences tumour initiation. Nature 2024; 633: 678–85. 39112713 10.1038/s41586-024-07797-z PMC11410659

[15] Brown ZJ, Tsilimigras DI, Ruff SM, et al. Management of hepatocellular carcinoma: a review. JAMA Surg 2023; 158: 410–20. 36790767 10.1001/jamasurg.2022.7989

[16] Ye LY, Chen W, Bai XL, et al. Hypoxia-induced epithelial-to-mesenchymal transition in hepatocellular carcinoma induces an immunosuppressive tumor microenvironment to promote metastasis. Cancer Res 2016; 76: 818–30. 26837767 10.1158/0008-5472.CAN-15-0977

[17] Dituri F, Mancarella S, Cigliano A, et al. TGF-β as multifaceted orchestrator in HCC progression: signaling, EMT, immune microenvironment, and novel therapeutic perspectives. Semin Liver Dis 2019; 39: 53–69. 30586675 10.1055/s-0038-1676121

[18] Ward FJ, Kennedy PT, Al-Fatyan F, et al. CTLA-4-two pathways to anti-tumour immunity? Immunother Adv 2025; 5: ltaf008. 40265076 10.1093/immadv/ltaf008 PMC12012449

[19] Naserkhaki R, Shokouhian B, Tahamtani Y, et al. Revisiting treatment strategies: addressing epithelial-to-mesenchymal transition-induced resistance in hepatocellular carcinoma. BME Front 2025; 6: 0144. 40556662 10.34133/bmef.0144 PMC12187357

